# Visualizing Non-abrupt Transition of Quantum Well States at Stepped Silver Surfaces

**DOI:** 10.1038/srep12847

**Published:** 2015-08-05

**Authors:** Srijan Kumar Saha, Sujit Manna, Valeri S. Stepanyuk, Jürgen Kirschner

**Affiliations:** 1 Max-Planck-Institut für Mikrostrukturphysik, 06120 Halle, Germany; 2 Martin-Luther-Universität Halle-Wittenberg, 06120 Halle, Germany

## Abstract

We use scanning tunneling spectroscopy (STS) experiments and first-principles density functional theory (DFT) calculations to address a fundamental question of how quantum well (QW) states for electrons in a metal evolve spatially in the lateral direction when there is a surface step that changes the vertical confinement thickness. This study reveals a clear spatially dependent, nearly continuous trend in the energetic shifts of quantum well (QW) states of thin Ag(111) film grown on Cu(111) substrate, showing the strongest change near the step edge. A large energetic shift equaling up to ~200 meV with a lateral extension of the QW states of the order of ~20 Å is found, even though the step-edge is atomically sharp as evidenced by a line scan. The observed lateral extension and the nearly smooth transition of QW states are understood within the context of step-induced charge oscillation, and Smoluchowski-type charge spreading and smoothing.

A variety of metal films grown on different substrates are known to exhibit electron confinement when potential barriers at the vacuum/film and film/substrate interfaces restrict the electron motion within the film^1^,^2^,^3^,^4^,^5^. As the thickness of the film is reduced to become comparable to electron’s de Broglie wavelength (*λ*_*d*_), the electrons moving along the direction perpendicular to the film surface become quantized into the well-known quantum well (QW) states^1^. The resulting two-dimensional (2D) electronic structures have intensively been studied in the past, mostly by laterally-averaging techniques such as photoemission(PES) and inverse photoemission sepectroscopy(IPES)^6^,^7^,^8^. A complementary approach is also opened by scanning tunneling microscopy (STM) and spectroscopy (STS)^9^,^10^,^11^, with lateral resolution on the atomic scale, where the information is not averaged over structural variations arising often due to surface defects such as steps, kinks and vacancies.

On any real surface of a solid, atomic steps are quite common. These topological features, which belong to a generic defect class, line defects, appear frequently during crystal growth and are known to play an important role in determining the pathways and reaction rates for heterogeneous processes such as chemical catalysis and corrosion^12^. Atomic steps and associated film thickness variations are very likely to have profound influence on the QW states due to step-induced Smoluchowski type charge smoothing effect^13^ as well as to modification of the local force fields in the region around steps. For films with such locally varying thickness, the question arises how the transition of the thickness-dependent QW states takes place and over what length scale. A further question of evident practical interest is whether it is possible to tune or modify the energy of these states laterally.

Motivated by these considerations, in this letter, we address the role of surface step on QW states in Ag overlayer films grown on Cu(111) substrate, using low temperature STS experiment combined with a first principles density functional theory calculation. This study reveals that the transition of QW state is more or less smooth with a certain lateral extension, contrary to the naive belief that the transition should be sharp and abrupt^14^ around steps as the well states are quantum in nature. We also find that the energy of these states has a strong distance dependence within the proximity of the step edge with large energetic shift equaling up to ~200 meV. For an Ag layer of 30.5 ML (monolayer) thick, we obtain experimentally a lateral extension of the QW states of the order of 20–24 Å. The pseudomorphic growth, well-defined interface, and atomically-resolved STS allow us to probe individual QW states arising from the electron confinement along the growth axis of Ag films and to determine the characteristic length scale of the QW transition, as these states are laterally highly localized and give rise to distinct and sharp peaks in the tunneling spectra. Our first-principles calculations in the framework of DFT account for the explanation of the observed effects and provide us with information on the characteristic length scale of quantum well transition.

Ag is a monovalent noble metal with fully filled 4d shell crystallizing in the fcc structure. For Ag, the bottom of the s-type valence band is situated below the binding energy of −7 eV and the top of the s-band above the Fermi energy (E_F_), leaving the energy range of −2.3 to −0.3 eV (where the QW states are observed in our experiment) for the bands with mostly p-type character (see Fig. 1). In this energy window, the band structures along the Γ-L(111) direction mainly consist of two bands : a highly dispersive parabolic 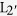 band originating mostly from p-states and a relatively flat band derived from *d*-states. Within the d-band region, two sharp peaks are located around binding energies of −3.8 and −5 eV. The 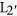 band touches E_F_ at the L point on the boundary of the first Brillouin zone, and decreases monotonically in energy towards the Γ point. There is an energy gap of about 4 eV at the L point above E_F_. When the Ag film becomes sufficiently thin along the growth axis direction (say, z) to realize the quantum confinement of Ag electrons in the film, the bands originating from sp-states are expected to form quantized states in the energy range of −2.3 to −0.3 eV. Indeed, for Ag(111) film, the QW states have been observed by photoemission over the widest range of thickness and binding energy for any overlayer/substrate combination hitherto studied^15^. For this reason and because of the metallurgical simplicity (Ag and Cu are completely immiscible at room temperature)^16^, we choose here the Ag/Cu(111) system for studying the interaction of QW states with the surface steps. An efficient experimental tool for studying the interaction of these QW states with surface steps is STM which probes the weak evanescent tails of QW electrons outside the metal in the vaccum region without destroying the interference pattern^17^.

The long diffusion length of Ag leads to the formation of large (111) like terraces with sharp rise to the next layer. Despite large lattice mismatch the basic structure appeares to be a closed packed Ag layer in parallel epitaxy; namely, Ag crystallographic axes are parallel to the corresponding Cu axes. Figure 2(a) displays an atomically resolved STM topography (45 Å × 45 Å) with a terrace edge corresponding to Ag film thickness of 30 ML and 31 ML. As expected the height difference between the two terraces is ~2.3 Å, corresponding to the ML height of the Ag film, as shown in height profile (top panel) of Fig. 2(b). The step edge is surprisingly sharp. The surface of both thicknesses exhibits a hexagonal unit cell consisting of Ag atoms with nearest neighbour distance 2.9 Å. STS differential conductance measurements across the step edge reveal well-defined states resulting from quantum confinement of sp-electron in the Ag film^18^. Figure 2(c) displays a series of tunneling spectra (dI/dV-V) measured on top of each atom along the line AB, across the step edge of 30 ML and 31 ML. The well-defined QW peaks are visible within the energy range of −2.3 to −0.3 eV near the Fermi level, indicating the presence of QW states (from n = 1 to 3) in this energy range. Ag(111) is also known to have pronounced surface state in the gap at the L-point. The location of the surface states can be strongly influenced by the strain effect (so they can be below E_*F*_ or above E_*F*_^19^). The peak around 0.2–0.3 eV could be the surface state, as identified in a similar STS study of Ag QW states on GaAs^20^. The formation of QW states modulates the density of states near E_*F*_. The changes of quantum well energy for each particular state across the step edge are measured very precisely. Far away (i.e. >1 nm) from the step edge, the tunneling spectra of two terraces are almost spatially independent (see Fig. 2(d)), i.e., the unperturbed peaks of two terraces are achieved. The two spectra correspond to film thickness of 30 ML and 31 ML, respectively. Previously, it was assumed that at the step edge both QW systems should exist without influencing each other, which was observed on a Pb wedge on Si substrate^14^. They showed that the spectrum measured at the step edge, fits perfectly with the calculated average of the n-ML and (n + 1)-ML spectra. However, we observed a different scenario in case of Ag films—a clear spatially dependent continuous trend in the energetic shifts of the QW states which shows the strongest change near the step edge. In contrast to the sharp step edge (as shown in line scan), a more or less smooth transition of QW energy with a lateral extension of 20–24 Å is observed. All energetic shifts for different QW are localized within a radius of ~10–12 Å from the centre of the step-riser region. This suggests that the lateral extension of QW energy is independent of the different QW states. Previously, for the transition of QW states at buried step edges^21^, the lateral change of the spectroscopic signal in the vicinity of the trenches appears as a continuous shift which coincides with the topographic height profile. In Ref. 21, a line scan perpendicular to the step edge shows that the step edge is not very sharp but a more or less smooth transition with a certain lateral extension. There topographical transition between the two terraces took place over a distance of 60 Å and as was shown for a particular QW state, the change of the QW energies across this transition is also continuous and occurs on the same length scale. This reference article clearly shows that the transition of QW states at step edges appears as a continuous shift which is consistent with the topographic height profile. In contrast, in our experiment, we observe a sharp step edge [see Fig. 2(b)]; and despite this sharp edge, a more or less smooth transition of QW energy with a lateral extension of 20–24 Å is found. The topography transition between the two terraces takes place over a distance of 5 Å as shown in Fig. 2(b). The change of quantum well energy for particular state across the step edges are determined precisely. The transition of QW states at step edges appears as a continuous shift and this continuous shift is not consistent with the sharp topographic height profile [compare Fig. 2(d) with 2(b)]. These experimental findings are indeed interesting and inspire us to perform a thorough ab-initio DFT calculation for a clear understanding of our experimentally observed effects.

The Ag(111) slab is represented by a supercell built from fcc Ag by stacking a variable number of fcc unit cells along the 111 direction and a Cu(111) substrate is modeled by continuing the fcc Ag lattice by Cu atoms [see lower panel of Fig. 3(a) and Fig. 4]. In the xy plane, which is the interface plane, the Cu lattice constant is adapted to the Ag value in order to avoid a lattice mismatch. The stepped Ag surface on Cu(111) substrate [for instance, Ag(4.5 ML)/Cu(111) system] is constructed by using a half-layer model where half of the atoms of one surface layer of a silver 111 oriented slab are removed. This leads to an Ag(111) overlayer slab with stepped top and flat bottom. In this structure, terraces form an infinite (along y-direction) stripe of Ag atoms, which is 15 atomic rows wide in the upper surface layer and 15 for the lower surface layer. This striped surface are five layers thick in cross-section through the upper terrace and four layers thick through the lower terraces (trenches) in between. Except the substrate (bottommost) layer, the rest of the whole system is relaxed so as to minimize the forces acting on the atoms using a conjugate-gradient algorithm. It is easy to conceive that the atoms near a surface step will suffer displacement from their (regular) flat (2D) surface lattice sites. Unlike flat surfaces, stepped surfaces relax in both z and x directions, since the existence of steps at the surface leads to broken symmetry in both of these directions. While relaxations along the z-direction yield modified interlayer separations, those along the x-direction provide new registries of atoms, as compared to those in the bulk. In contrast to bulk atoms the forces acting on surface atoms near step are unbalanced leading to resultant forces and hence, to a geometrical relaxation (in addition to the relaxation of the surface atom layer as a whole). This relaxation can further unbalance the forces acting on the next-nearest neighbors and so forth, giving rise to an elastic strain emanating from the step site.

The relaxed structure of Ag(4.5 ML)/Cu(111) system is rendered in Fig. 3 (top-view) and Fig. 4 (side-view). A more reduced number of bonds (lower coordination) at step edeges results in a shorter in-plane (along x-direction) interatomic distances. As the coordination number decreases (from atom 8 to 1 for upper terrace), the bond-length (Δ*x*_*i*+1,*I*_ = *x*_*i*+1_ − *x*_*i*_, where *i* stands for atom index) decreases [see Fig. 3(b)]. In the region near the step base, the interaction energy is stronger (i.e., the total energy is lower) as the number of nearest neighbors is increased. The reverse argument suggests an increased activation barrier for crossing the upper step edge. And this lowering of the surface potential turns out to be confined around the step, because already the second row of Ag atoms away from the step gives the height profile (interlayer distance) characteristic for the (unperturbed) terrace [see Fig. 2(b) and Fig. 3(a)], which by the way is the same as for the respective flat (unstepped) surface.

These elastic deformations are accompanied by changes of the local electronic behaviour (or vice versa). As electronic states are more delocalized and take a longer distance to make the transition than lattice, a smoothing and spreading of the electron cloud at surface step should occur^13^,^22^. This Smoluchowsky-type smoothing effect of the electron density causes a charge dipole at the step site, with the appearance of a more positive charge at the top of the step, and a more negative charge close to the step base. The step dipole has a surface normal component opposite in direction to the usual charge spreading at the metal surface caused by the spillover of the electron cloud into the vacuum and as a result will reduce the effective potential at the step base site.

This charge redistribution, in turn, is another driving force for the inward relaxation of the step atoms. For the Wigner-Seitz cells of the step atoms charge neutrality is no longer conserved. The charge flow to the lower terrace leaves these cells positively charged and with a rather high self-energy. This self-energy can be lowered if the nuclei of the step atoms (at upper terrace) are displaced toward the region of higher charge density, that is inward. The opposite is true for the atoms at the step-base (at lower terrace). As a whole the step is “rounded off” [see Fig. 2(b) and Fig. 3(a)].

In addition to the Smoluchowski-type electron smoothing, electrons near a step attempt to screen the defect represented by the step, giving rise to defect-induced Friedel-type oscillations^23^,^24^. This means that the effects on the electronic structure of the terrace caused by the step are confined to a region of the order of its screening length (~20 Å) which determines the range of the step-induced alteration of the film properties.

We find that the fifth row of Ag atoms away from the step already gives the energy peaks characteristic for the (unperturbed) terraces (Fig. 4, upper panel), which are the same as for the respective flat (unstepped) films. This lateral extension is, in fact, larger than the length-scale associated with the height profile (nuclear) modification (i.e, the second row of Ag atoms as mentioned above). The insets of Fig. 4 show the step-induced electron oscillation modified by the effect of Smoluchowski-type charge spreading and smoothing. The insets also show that the upper (5 ML) terrace exhibits larger amplitudes of oscillation than the lower (4 ML) terrace which can be attributed to the fact that the upper terrace has lower atomic coordinations than the lower terrace. For this reason, this electron oscillation on one side is asymmetric in nature relative to the other side near the step edge. These oscillations, when their amplitudes are small, are not observed in our experiment due to possible thermal and other noises. If this electron oscillation was the only dominant effect near step-edge then one must get a sharp abrupt transition. However, in reality, the effect of Smoluchowski-type charge spreading and smoothing becomes much stronger near the step-edge and make the transition of QW states more or less smooth and continuous (see Fig. 4). The fitted line (dashed) in Fig. 4, is very similar to the experimental fit [see Fig. 2(d)]; which clearly suggests that the combination effect of step-induced charge smoothing and of charge screening should be prudently considered.

In conclusion, we use STS experiments and first-principles DFT calculation to investigate the role of steps on QW states in Ag overlayer films grown on Cu(111) substrate. This study reveals that the transition of QW state is nearly smooth with a certain lateral extension, and the energy of these states has a strong distance dependence within the proximity of the step edge with large energetic shift equaling up to ~200 meV. For an Ag layer 30.5 ML thick, we obtain a lateral extension of the QW states of the order of 20–24 Å. This lateral extension of QW states is understood within the context of induced screening and smoothing of electrons near step edges. These findings suggest a real particle-in-a-tunable-box system which would be of great value for basic studies of quantum phenomena as well as hold out the promise of device applications using the high-mobility electrons found in metallic systems.

## Methods

### Theory

All our calculations are performed using the VASP^25^ implementation of DFT, with the Perdew-Burke-Ernzerhof exchange-correlation functional for solid (PBEsol^26^) and projector augmented wave (PAW) potentials^27^,^28^. Kohn-Sham wave functions are represented using a plane-wave basis truncated at an energy cutoff of 40 Ry. The Brillouin zone integrations are done on a uniform Monkhorst-Pack^29^ **k** grid of 19 × 19 × 19 for the bulk and 19 × 19 × 1 for the slab calculations.

### Experiment

Our experiment was carried out in an ultra-high vacuum (UHV) chamber with base pressure below 5 × 10^−11^ mbar. The Cu(111) single-crystal substrate was cleaned with several cycles of sputtering with 1 keV Ar^+^ sputtering and subsequent annealing at 780 K. The deposition of Ag was performed by evaporation from a tungsten crucible heated by a feedback controlled electron beam in a wedge shape with a slope of ~3 ML/min. The substrate temperature during Ag deposition was about 373 K. The sample was allowed to cool down to room temperature before it was transferred to the cold STM chamber. The chemical cleanliness, growth and surface morphology of the substrates and films were verified by Auger electron spectroscopy (AES), low energy electron diffraction (LEED), reflection high energy electron diffraction (RHEED) and STM. All STM/STS measurements were done at 4.7 K using chemically etched polycrystalline W tips followed by flashing the tip to ~2200 K. The differential conductivity 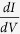 was measured in STS mode using a lock-in technique with a typical modulation frequency of 32.8 kHz and modulation voltage of 10–15 mV. The spectra were taken in both directions, from higher to lower and from lower to higher sample bias, in order to correct for energy shifts due to finite time constant.

## Additional Information

**How to cite this article**: Kumar Saha, S. *et al.* Visualizing Non-abrupt Transition of Quantum Well States at Stepped Silver Surfaces. *Sci. Rep.* **5**, 12847; doi: 10.1038/srep12847 (2015).

## Figures and Tables

**Figure 1:**
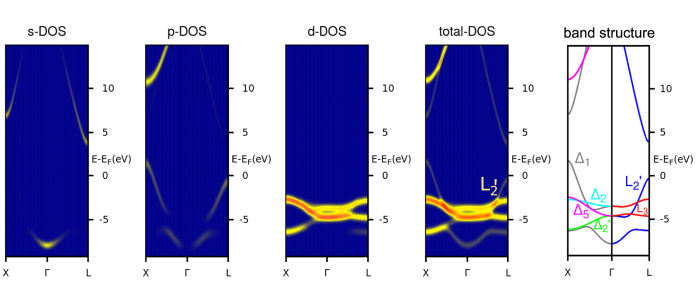
Orbital selective k-resolved electronic density of states EDOS of bulk Ag along with its band structure. The 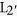 band mostly has *sp*-orbital character responsible for our observed QW states. The color scale yellow (light) and red (dark) correspond low and high EDOS respectively.

**Figure 2:**
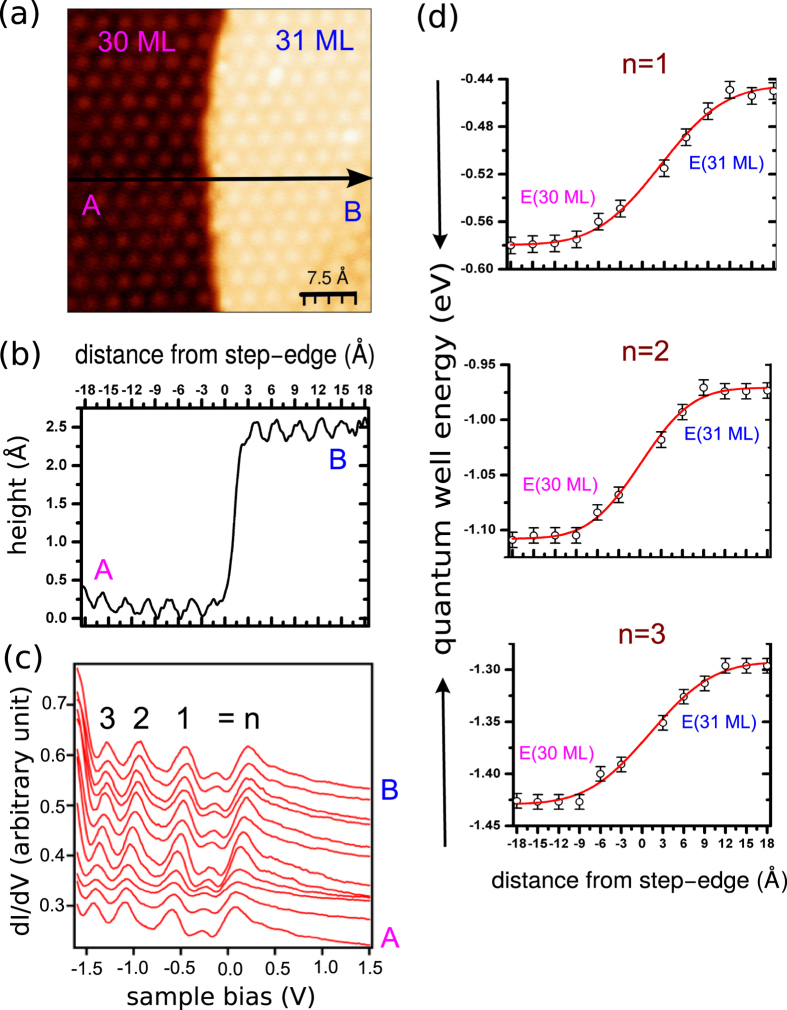
(**a**) Atomically resolved STM topography (45 Å × 45 Å) with terrace edge corresponds to Ag film thickness of 30 ML and 31 ML. (**b**) STM height profile across the step edge. (**c**) A series of tunneling spectra (dI/dV) measured on top of each atom along the line AB, across the step edge. (**d**) The energy position of the various QW states with respect to the distance from the step edge.

**Figure 3:**
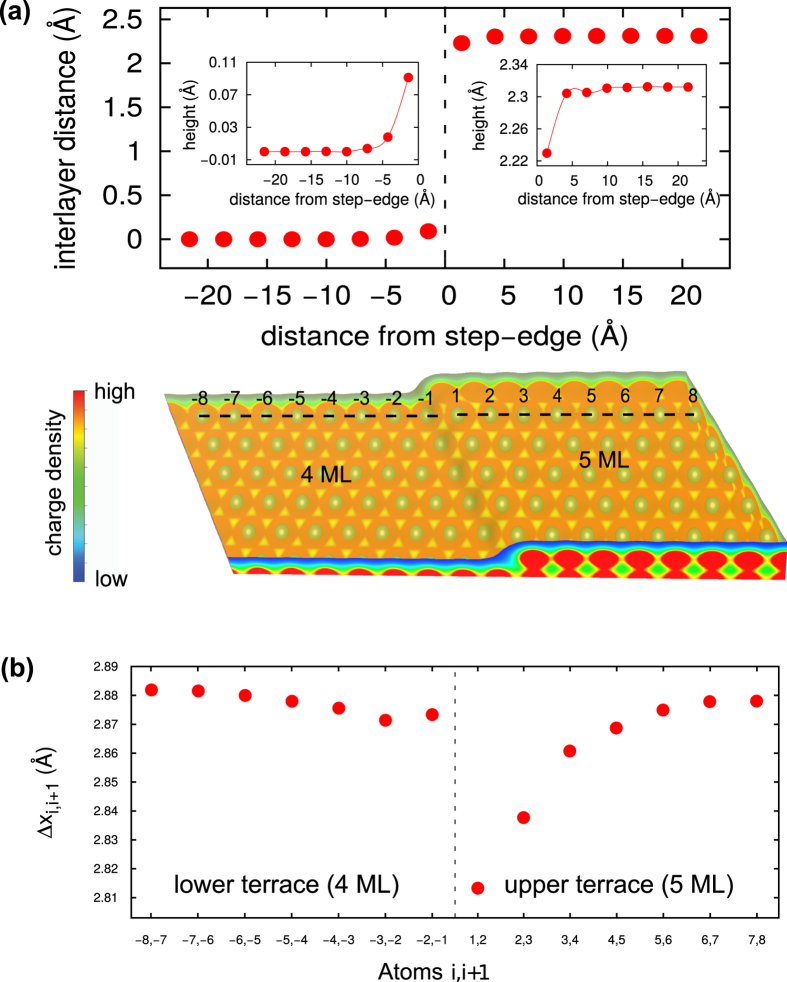
(**a**) Lower Panel : A linescan taken across the step indicated by dashed black line. Upper Panel: Calculated (DFT) height profile across the step for this linescan. (**b**) Calculated in-plane (along x-direction) bond-lengths (Δ*x*_*i*+1,*I*_ = *x*_*i*+1_ − *x*_*i*_, where *i* stands for atom index) across the step for this linescan.

**Figure 4:**
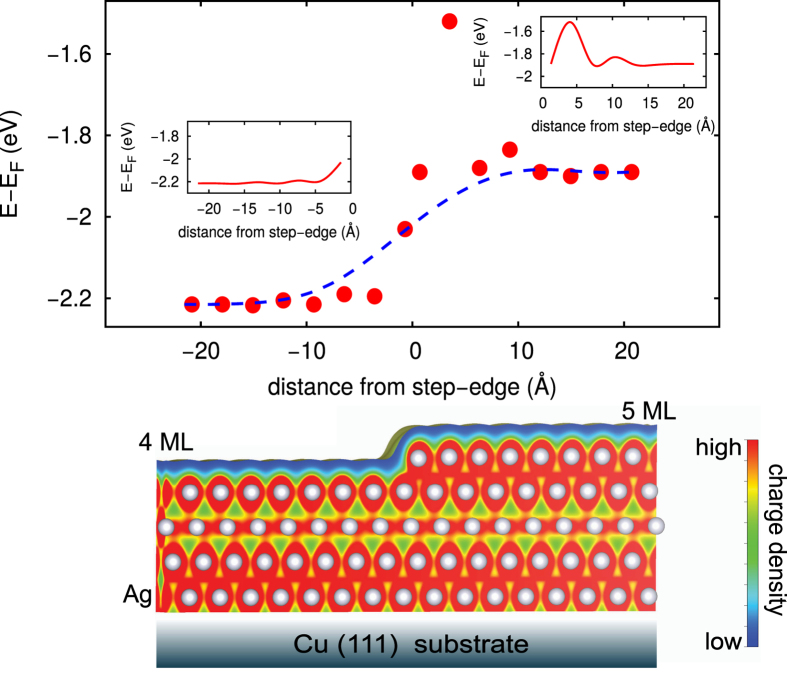
The energy position of an individual QW (DFT) peak as a function of distance from the step-edge. Red dots are the positions of the above peak in the surface atom-resolved theoretical electronic density of states. The insets show the step-induced (line defect) electron oscillation (Friedel-type) modified by the effect of Smoluchowski-type charge smoothing. The Bézier-fitted line (dashed) is very similar to the experimental fit [see Fig.2(d)], which indicates that the transition of QW state is nearly smooth with a lateral extension of ~±12 Å. A side-view of the relaxed structure of Ag(4.5 ML)/Cu(111) system is rendered at lower panel.
